# Improved Method for the Quantification of Motility in Glia and Other Morphologically Complex Cells

**DOI:** 10.1155/2013/853727

**Published:** 2013-11-20

**Authors:** Mari Sild, Robert P. Chatelain, Edward S. Ruthazer

**Affiliations:** ^1^Montreal Neurological Institute, 3801 University Street, McGill University Montreal, QC, Canada H3A 2B4; ^2^Department of Psychiatry, McGill University, Canada; ^3^Department of Physics, McGill University, Canada

## Abstract

Cells such as astrocytes and radial glia with many densely ramified, fine processes pose particular challenges for the quantification of structural motility. Here we report the development of a method to calculate a motility index for individual cells with complex, dynamic morphologies. This motility index relies on boxcar averaging of the difference images generated by subtraction of images collected at consecutive time points. An image preprocessing step involving 2D projection, edge detection, and dilation of the raw images is first applied in order to binarize the images. The boxcar averaging of difference images diminishes the impact of artifactual pixel fluctuations while accentuating the group-wise changes in pixel values which are more likely to represent real biological movement. Importantly, this provides a value that correlates with mean process elongation and retraction rates without requiring detailed reconstructions of very complex cells. We also demonstrate that additional increases in the sensitivity of the method can be obtained by denoising images using the temporal frequency power spectra, based on the fact that rapid intensity fluctuations over time are mainly due to imaging artifact. The MATLAB programs implementing these motility analysis methods, complete with user-friendly graphical interfaces, have been made publicly available for download.

## 1. Introduction

The development of advanced imaging methods has revealed that living cells exhibit highly dynamic structural remodeling [1, 2]. The rate and structural details of cellular motility can change over development or in response to the environment in ways that reveal important details about cellular signaling mechanisms [3–5]. Useful commercial and public domain programs are available to identify and measure the movements of relatively large cell structures like amoeboid pseudopodia [6–8] as well as fine features like growth cone filopodia [9], astrocytic protrusions [10], and dendritic spines [11, 12]. Analysis of these kinds of sparsely distributed cellular protrusions may be amenable to the use of image skeletonization or active contour tracing to select the features of interest and to track end-tips for motility analysis. An alternative approach is to measure perimeter shape changes of an entire cell by analyzing the difference between an original image and an “eroded” image from which all fine processes have been filtered away [10]. However, if the features of interest are very dense and spindly, overlapping and oriented in many directions as in the case of astrocytes and radial glia [13, 14], quantitative measurements of motility become particularly challenging for any of the above methods. 

 We have previously introduced a method for quantifying motility in cells with densely packed, fine processes as part of a study analyzing dynamic remodeling of midbrain radial glia [15]. After collecting a time series of 2-photon z-stacks, maximum intensity projections (i.e., maximum intensity value at each *x*-*y* position through the z-stack is used to generate a 2D projection) are aligned to correct for drift and then images are preprocessed by binarization using Sobel edge detection, followed by pixel dilation to remove gaps generated by the edge detection procedure. The algorithm then calculates a motility index, defined as the mean number of redistributed (added or lost) pixels between processed images at sequential time points. To deal with the wide range of different cell sizes, indices are normalized for size by dividing by cell area (in pixels). 

One potential limitation of this approach is that normalizing pixel counts to the 2D projection area of each cell can distort the results due to variations in cell shapes and sizes. For example, z-projections of very long, narrow cells like radial glia will give dramatically different measurements of 2D projected area depending on whether a cell is viewed parallel or perpendicular to its radial axis. Although this index is still highly sensitive for measuring relative changes in motility of individual cells imaged from the same angle over time in response to different treatment conditions, the area normalization used to calculate the motility index introduces a source of artifact that limits its usefulness for comparing multiple cells that might be imaged at a range of different orientations. 

In this paper, we introduce several significant improvements to our original method that make important advances toward correcting these limitations. The new approach offers greater sensitivity for cell motility analysis while decreasing the impact of total cell area on measurements by using an algorithm based on spatial boxcar averaging of redistributed pixels. This new motility index provides a value that better correlates with the mean elongation and retraction speeds of moving filopodial processes, eliminating the need to normalize by total cell area. Boxcar averaging measures motility preferentially in the dynamic parts of the image by giving more weight to pixel redistributions that occur in close proximity to other redistributions and are thus more likely to be part of real restructuring events. In addition, for high-temporal-resolution imaging, we have added a noise filter based on the temporal frequency power spectra of the pixels, optimized to preserve biological activity while removing imaging artifacts. We present these new tools as part of an open-source program for the popular MATLAB platform with a customizable user interface that can easily be applied to measure motility of a wide variety of cell types and structures. 

## 2. Materials and Methods

### 2.1. Imaging EGFP-F-Transfected Radial Glia Cells

**Figure 1:**
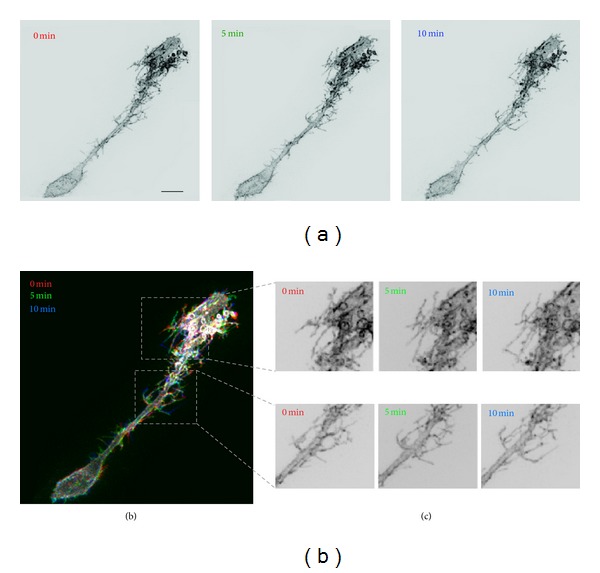
Radial glia process movement *in vivo* is apparent over a timescale of minutes. (a) Maximum intensity projections of two-photon microscope z-series images of a radial glia cell expressing farnesylated EGFP in the intact tadpole brain. (b) RGB overlay of the time-lapse images of a radial glia cell where red denotes the position of the cell at 0 minutes, green after 5 minutes, and blue after 10 minutes of imaging. (c) Magnified images of several highly dynamic sites from the radial glial cell shown in (b). Scale bar = 10 *μ*m.

The cells examined for this study are radial glia in the optic tectum of albino Xenopus laevis tadpoles at developmental stage 47. Tadpoles were staged according to the criteria of Nieuwkoop and Faber [16]. Radial glia have many fine filopodial processes that undergo continuous structural remodeling over the timescale of minutes (Figure 1). The optic tectum in stage 45 tadpoles was bulk electroporated [17] with a DNA plasmid encoding membrane-targeting farnesylated EGFP (EGFP-F). Tectal radial glia cells were imaged two days later using a custom-built two-photon laser-scanning microscope with a 60 × 1.1 NA water-immersion objective (Olympus). A MaiTai-BB femtosecond pulsed Ti : sapphire laser (Newport/Spectra Physics) was used to generate excitation light at 910 nm. In preparation for imaging, tadpoles were paralyzed by immersion in 0.2 mM pancuronium bromide (Sigma) solution in 0.1x Modified Barth's solution with HEPES (VWR). Images of individual EGFP-F-expressing cells in live albino tadpoles were collected as z-series stacks of 50–150 optical sections sized 512 pixels × 512 pixels (79.5 *μ*m × 79.5 *μ*m) *μ*m at 1 *μ*m step size. In a typical experiment, 3 images were collected at 5 min intervals as the baseline. Subsequently, MK-801 maleate (100 *μ*M, Tocris) was injected intraventricularly into the tadpoles at the level of the optic tectum. After a 20 min incubation, another 3 images were captured at 5 min intervals to assess the effects of the treatment on motility. In another set of experiments to estimate the contribution of sample drift and noise artifact, animals were euthanized in 0.2% MS222 (Sigma) and fixed by immersion in 4% paraformaldehyde (PFA, VWR) for 2 hours prior to imaging. 

### 2.2. Image Registration for Analysis

Two-photon z-series stacks were processed by blind deconvolution using AutoQuant software. As all images of cells were captured *in vivo*, surrounding neurons and skin cells expressing EGFP as well as autofluorescent melanophores were sometimes present in a subset of z-series images, above or below the cell of interest where they could interfere with the automatic motility assessment. These non-glia objects were removed manually using ImageJ from the z-series stacks [18]. The careful removal of extraneous objects from the stacks is critical for obtaining accurate measurements and typically takes about 30 minutes per 100 optical sections. 

The inherent voxel anisotropy in 2-photon microscopy—axial resolution is always poorer than resolution within the focal plane—makes perfect 3D registration of image stacks impractical. We therefore designed our motility analysis for 2D time series and took precautions to minimize rotation during image acquisition. Tadpoles were mounted in form-fitting polydimethylsiloxane (PDMS) imaging chambers sealed with a coverslip to minimize rotation of the samples over time. Z-series stacks were converted to 2D maximum intensity projections and realigned to correct for slight lateral drift over time using the rigid body setting of the EPFL StackReg plugin for ImageJ (http://bigwww.epfl.ch/thevenaz/StackReg/) [19]. 

### 2.3. Analysis of Morphological Dynamics

All subsequent processing and analysis steps in this paper were performed using custom-made scripts that can be run under the MATLAB (MathWorks) platform. These routines have been made available under a modified version of the GNU General Public License v3 (see details in program files) as part of a set package controlled with a user-friendly graphical user interface available at http://ruthazerlab.mcgill.ca/downloads/motility_GUIv2.zip.

**Figure 2:**
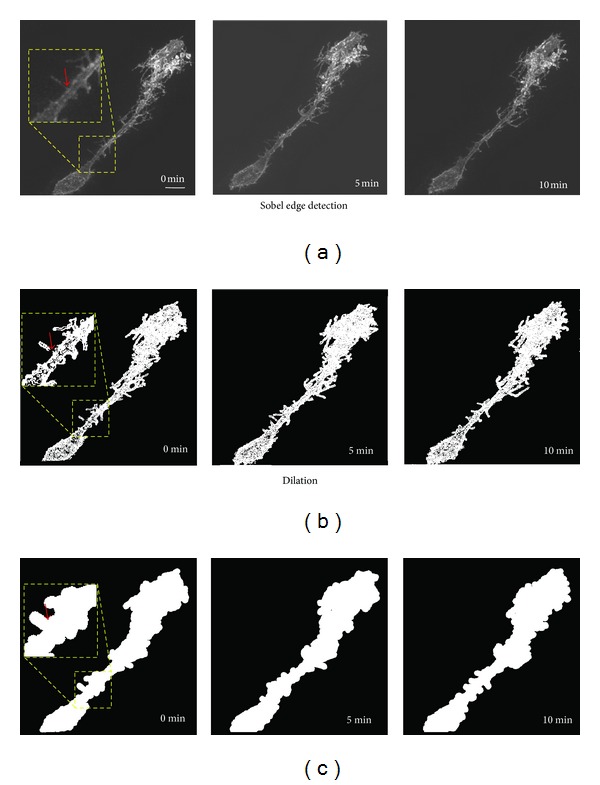
Image preprocessing for analysis. (a) Maximum intensity projections of two-photon images of a radial glia cell expressing farnesylated EGFP *in vivo* collected at 0, 5, and 10 minutes. (b) Same images after Sobel edge detection and binarization. (c) Binarized images underwent dilation with a 6-pixel radius. Pixel dilation helps eliminate artifactual discontinuities in the images (arrowhead in insets) resulting from the binarization step. Scale bar = 10 *μ*m.

After optimizing the alignment of the maximum intensity projections for each time point as described above, images were subjected to preprocessing by binarization using Sobel edge detection to identify edges, followed by pixel dilation (Figure 2). Pixel dilation expands the edges of the binarized images, largely eliminating discontinuity artifacts that sometimes occur in the edge detection step due to variations in the pixel intensities at fine processes in the raw images (e.g., arrowhead in Figure 2). A dilation radius of 6 pixels was used, as this was determined empirically to remove this artifact efficiently while still maintaining adequate resolution to detect small movements of the cell surface.

**Figure 3:**
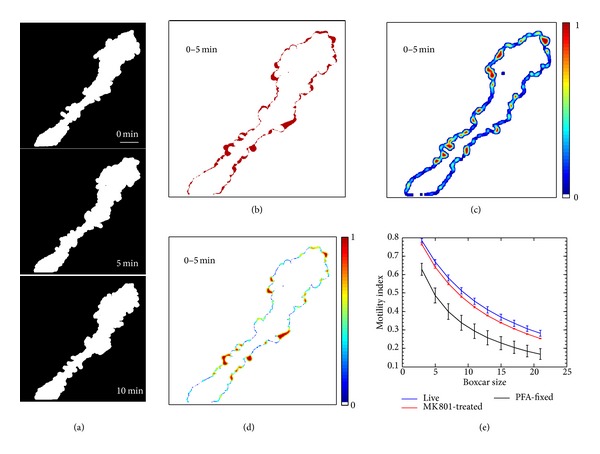
Analysis of pixel redistribution using boxcar averaging. (a) Preprocessed images from 0, 5, and 10 minutes time points. (b) Subtracting consecutive images from one another creates a map of the redistributed pixels between time points, shown in red. (c) Boxcar averaging of the pixel redistribution images from (b) using 9 × 9 pixel window. (d) Result of multiplication of the images in (b) and (c). The average of the nonzero pixel values in this image is used to calculate the cell motility index. (e) Plot of motility index values obtained for the datasets in Figures 5(c), 5(d), 6(c), and 6(d) using a range of different boxcar sizes. Motility values in the different conditions are most clearly separated when a 9 × 9 pixel boxcar size is used. Scale bar = 10 *μ*m.

To assess the motility of the cell we subtracted the processed images of consecutive time points (Figure 3(a)) from one another. We define the binary intensity value of an image with dimensions *x* and *y* as *I*_*xy*_(*t*) where *t* is the time index of the image ranging from 1 to *T* in integral steps, with *T* being the number of time points captured. The redistribution of pixels between two time points, for each pixel defined by (*x*, *y*), is calculated as

(1) $$\begin{array}{c} \delta_{xy}\left(t\right)=I_{xy}\left(t+1\right)-I_{xy}\left(t\right). \end{array}$$

The absolute value of this subtraction result generates an image highlighting pixels where a change has taken place from one time point to another, showing pixel redistribution (Figure 3(b)). In the original dynamics assessment approach of Tremblay et al. (2009), which we here term Method 1, the average total area of the images is taken into consideration. The area of an image with time index *t* is defined by

(2) $$\begin{array}{c} A\left(t\right)=\overset{}{\underset{x,y}{\sum }}I_{xy}\left(t\right). \end{array}$$

Sum of the redistributed pixels between time points is calculated:

(3) $$\begin{array}{c} R\left(t\right)=\overset{}{\underset{xy}{\sum }}\delta_{xy}\left(t\right). \end{array}$$

Motility index *M*_1_ between two time points using Method 1 is defined as

(4) $$\begin{array}{c} M_{1}\left(t\right)=\frac{R\left(t\right)}{\bar{A}}\;, \end{array}$$

where $\bar{A}$ is the average total area of the cell over all time points.

In the new, improved algorithm, here referred to as Method 2, the difference images calculated by formula (1) are subjected to boxcar averaging, often referred to as taking a “moving average”, (Figure 3(c)) as defined by

(5) $$\begin{array}{c} {\hat{\delta }}_{xy}\left(t\right)=\frac{1}{w^{2}}\overset{x+h}{\underset{m=x-h\;\;}{\sum }}\overset{y+h}{\underset{n=y-h}{\sum }}\delta_{mn}\left(t\right), \end{array}$$

where *w* denotes the boxcar window width and *h* = (*w* − 1)/2. The choice of the boxcar window size for averaging should be determined empirically depending on the dimensions of the structures imaged, taking into consideration that important details may be lost as the boxcar size is expanded. We set the boxcar size to *w* = 9 (9 × 9 pixel square) for our analysis. Next, the boxcar-averaged image (Figure 3(c)) is multiplied with the image showing redistribution of pixels between time points (Figure 3(b)) to obtain a final boxcar-weighted image *θ*_*xy*_(*t*) (Figure 3(d)):

(6) $$\begin{array}{c} \theta_{xy}\left(t\right)={\hat{\delta }}_{xy}\left(t\right)\delta_{xy}\left(t\right). \end{array}$$

This last step has the useful effect of blunting the contributions of the large number of sparse individual pixels found along the edges of the boxcar-averaged images, thus weighting the analysis in favor of dynamic “hot spots” where large groups of pixels change over time. These highly dynamic zones are expected to reflect biologically relevant pixel redistributions whereas the sparse intermittent changes along the periphery are more likely to arise from nonbiological artifact such as specimen drift, photobleaching, and detector noise.

Finally, the mean pixel value *M*_2_ of the filtered boxcar averaged pixel redistribution image *θ*_*xy*_(*t*) is calculated by

(7) $$\begin{array}{c} M_{2}\left(t\right)=\frac{1}{N}\overset{}{\underset{x,y}{\sum }}\theta_{x,y}\left(t\right), \end{array}$$

where *N* is the count of nonzero pixels. We thus define *M*_1_ and *M*_2_, respectively, as the original and improved motility indices measured between two time points. For the overall motility value of a cell under a certain condition, the mean motility index $\bar{M_{1}}$ or $\bar{M_{2}}$ over all time points was used.

The size of the boxcar window used for analysis is a customizable parameter in our software. The optimal setting depends in large part on the dimensions of the most motile processes extending out from the cell and the density with which they occur at the cell surface. We recommend using an independent dataset to empirically determine the parameters that give the greatest sensitivity for the cell type being studied. For example, in Figure 3(e), we systematically varied the boxcar window size and found that although the absolute motility index values decrease with larger boxcar sizes, the greatest difference between experimental groups is obtained with an intermediate boxcar size, in this case 9 pixels. 

### 2.4. Temporal Frequency-Based Filtering of Spurious Motility

**Figure 4:**
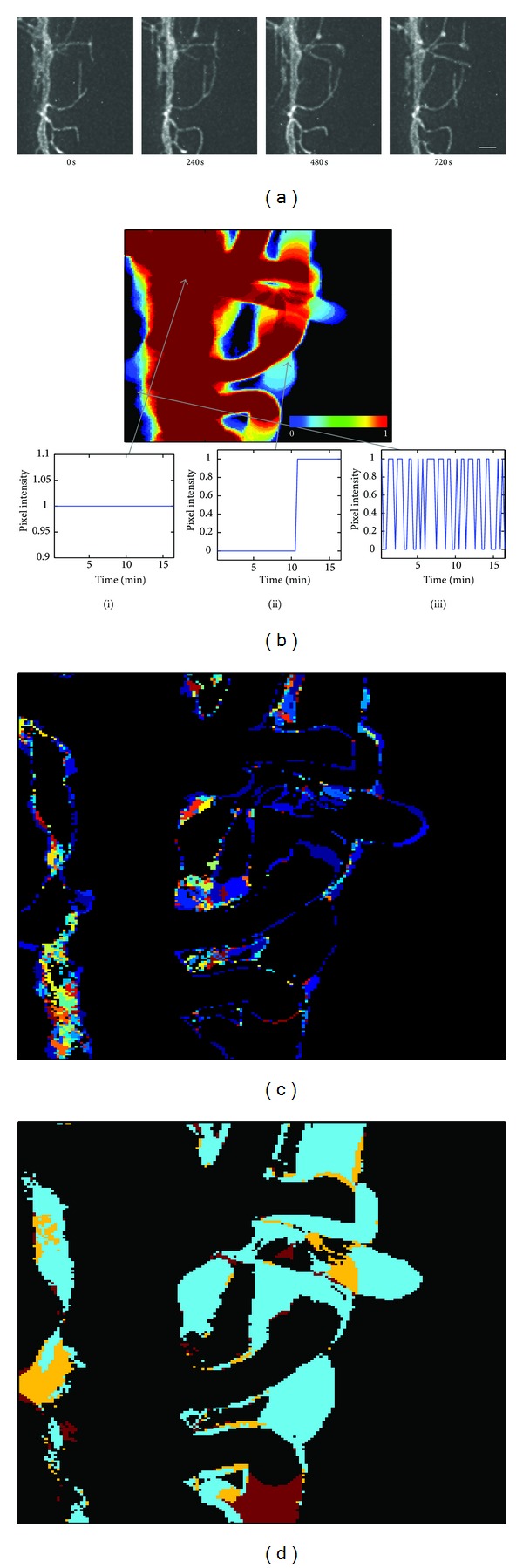
Temporal frequency filtering helps exclude artifactual pixel redistributions. (a) Time series of two-photon images of a portion of a radial glia cell. Scale bar = 2 *μ*m. (b) Integrated intensity of a healthy cell over the course of 18 minutes, preprocessed for analysis. Red pixels have a value of 1 during most time points and blue pixels during few time points. Black pixels are negative background sites with value 0 at all time points. Time-dependent pixel intensity functions (i) for a pixel located within the main process, (ii) for a pixel into which a filopodium extended, and (iii) for a noisy pixel located along the edge of the cell. (c) Frequency map showing noisy pixels that were discarded by temporal frequency filtering. The colors represent discrete high frequencies measured in the excluded range. (d) Frequency map of the same cell, showing pixels that were accepted by the frequency filter. Each color shows a discrete frequency within the accepted range. In (c) and (d), black represents sites where pixel intensity did not change over the 18 minutes.

In another experiment, images of an active cell were taken at the higher rate of once every 20 seconds over the 15 to 18 minutes normally used for dynamics analysis (Figure 4(a)). This sampling rate (0.05 Hz) constitutes temporal oversampling for the cellular motility behavior in which we are interested. For technical reasons only a subsection of the cell was measured in order to achieve the necessary temporal resolution. Images were preprocessed as described above, resulting in dilated binary images as in Figure 2(c). Three characteristic examples of the time-dependent behavior at single pixels are shown in Figure 4(b). Pixels located at the center of the cell main process typically will have an unvarying intensity function as shown in Figure 4(b)(i). Similarly, pixels that lie within the trajectory of an elongating or withdrawing filopodium will be continuously on or off until the filopodium either moves into or out of the pixel position, after which the pixel value will flip (Figure 4(b)(ii)). Pixels that are near the edge of the cell tend to alternate between a value of 1 and 0 irregularly, due to imaging artifact and alignment-drift error (Figure 4(b)(iii)). The time-domain pixel intensity functions shown were transformed into the frequency domain by means of the discrete fast Fourier transform (DFT) algorithm of MATLAB. A large proportion of the spurious motility can be filtered based on the DFT results, as image noise is expected to have a higher frequency DFT peak than biologically relevant slower fluctuations. We therefore implemented a DFT-based image filter with a user-selectable frequency threshold as part of our analysis package. Setting the filter to separate fluctuations at a threshold of 0.0026 Hz (<6.4 min periods) appeared to effectively distinguish between image noise at high frequency (Figure 4(c)) and biologically relevant motility fluctuating at lower frequencies (Figure 4(d)).

## 3. Results

To assess our improved method for cell morphological dynamics measurement, we electroporated albino Xenopus laevis tadpoles with plasmid driving the expression of membrane-targeting farnesylated EGFP (EGFP-F). Two days after electroporation, we collected images of individual EGFP-expressing radial glia cells in the optic tectum in live tadpoles by two-photon microscopy.

**Figure 5:**
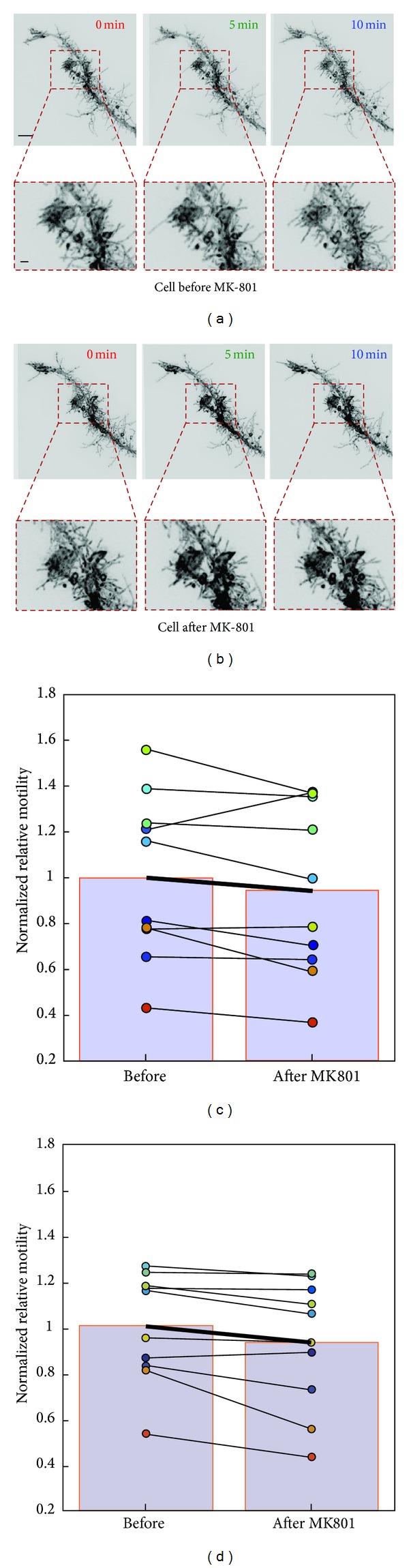
Comparing the relative sensitivity of the two motility measurement methods using images of normal radial glia cells (*N* = 10) before and after injection of 100 *μ*M MK-801 into the ventricle of the tadpole to reduce motility. ((a), (b)) Example of glial motility before (a) and after (b) drug treatment. (c) Motility index changes measured using Method 1 from [15]. (d) Results using the boxcar-based Method 2. Each individual cell is depicted with the same color in (c) and (d). Note that Method 2 gives more consistent results. All values are normalized to the mean predrug motility index to facilitate comparison. Scale bar = 10 *μ*m in upper panel and 2 *μ*m in lower panel of (a).

We tested the sensitivity of our improved motility index (Method 2) compared to the original algorithm (Method 1) from Tremblay et al. (2009), using an established pharmacological manipulation known to reduce radial glial motility. We have previously shown that radial glia decrease their filopodial process motility *in vivo* in response to intraventricular application of the N-methyl-D-aspartate receptor (NMDAR) blocker MK-801 [15]. Here we used this glial response to MK-801 to compare the relative sensitivity of Methods 1 and 2. In the study by Tremblay et al., time-lapse images had been collected at 15 minute intervals. In order to push the limits of our detection capability, we shortened the imaging interval to 5 minutes (Figures 5(a) and 5(b)). The cumulative restructuring of the cells is considerably less during this shorter interval and we sought to determine whether either analysis method could still detect a difference between control and MK-801-treated conditions. 

Tectal radial glia are highly dynamic on a timescale of minutes, continually adding and retracting many filopodial processes. However, although they remodel extensively, they do not exhibit cumulative growth over these periods. We found no significant difference between the area profiles of radial glia cells before compared to after MK-801 treatment (area mean ± SD before MK-801: 1465.25 ± 140.56 *μ*m^2^, after MK-801: 1411.36 ± 150.6 *μ*m^2^, *n* = 10, *P* = 0.45, two-tailed Student's *t*-test). We were unable to demonstrate a significant decrease in the motility index values of cells in response to MK-801 application when imaged at 5 min intervals and analyzed using Method 1 (*n* = 10, *P* = 0.11, paired *t*-test) (Figure 5(c)). In contrast, applying Method 2 to the same dataset revealed a significant decrease in the rates of dynamics of these cells after MK-801 treatment (*n* = 10, *P* < 0.02, paired *t*-test) (Figure 5(d)). The greater sensitivity of Method 2 was likely due to smaller variance in the motility index values resulting from the reduced contribution of artifact from nondynamic parts of the cell.

**Figure 6:**
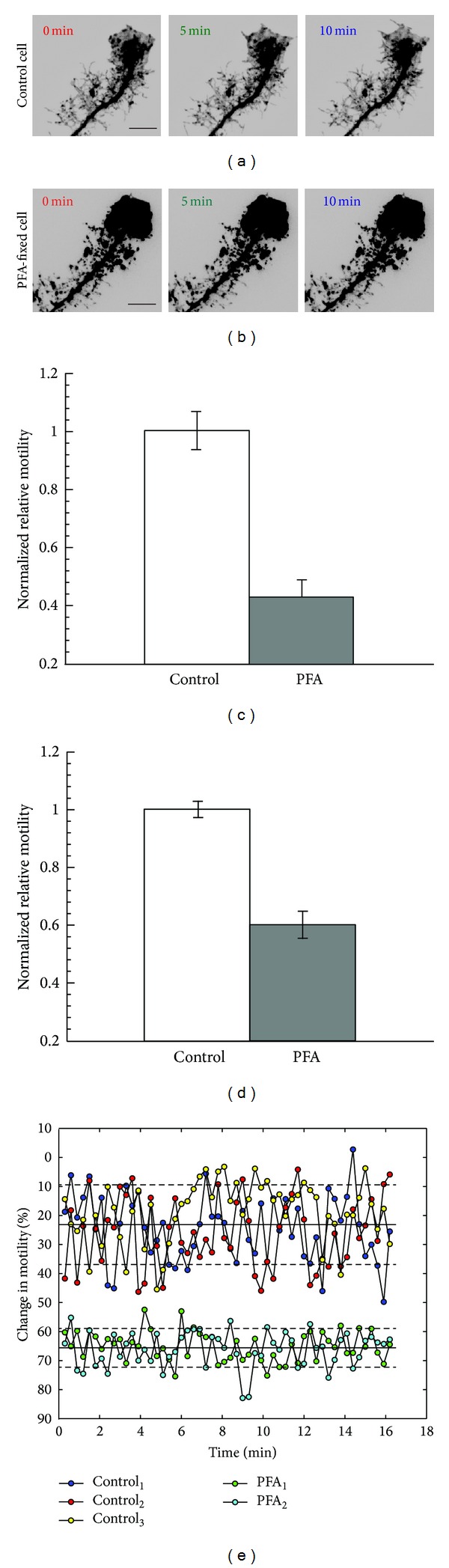
Use of paraformaldehyde- (PFA-) fixed cells to reveal nonbiological artifacts that contribute to the motility index scores. (a) The motility of control living cells (*N* = 4) was compared with (b) dead cells fixed in paraformaldehyde (*N* = 5). ((c), (d)) Bars indicate the motility values of the two groups normalized to the mean of control values using Method 1 in (c) and Method 2 in (d). Error bars are SEM. (e) Applying a low-pass temporal frequency filter with a threshold of 0.0026 Hz (6.4 min period) reduces the motility index scores (measured using Method 2) of the PFA-fixed cell group by substantially more than the control group, indicating that the filter is effective at eliminating nonbiological noise. Dashed lines indicate full width at half maximum. Scale bars = 10 *μ*m.

To assess the relative contributions of spurious motility caused by imaging artifact, we compared motility index values of control EGFP-F-expressing radial glial cells in living tadpoles (*n* = 4) with cells from the animals that had been euthanized and fixed with 4% PFA (*n* = 5) (Figures 6(a) and 6(b)). Fixation abolishes all cellular dynamics, leaving only imaging artifact, photobleaching, and Brownian motion of the fluorophore molecules. Not surprisingly, a large difference in cell motility scores between the control and fixed groups was obtained using both the old and new analysis methods. Somewhat unexpectedly, motility index scores obtained using Method 1 (Figure 6(c)) were on average lower, after normalization to the matched control group, than those produced by Method 2 (Figure 6(d)), probably reflecting a greater sensitivity of the new method to systematic artifact due to photobleaching or specimen drift. However, Method 1 exhibited larger variance in the motility indices obtained and thus gave a wider 95% confidence interval for the difference between means (CI = [0.3589, 0.7854]). Analysis by Method 2 resulted in a smaller two-sided 95% confidence interval for the difference between means (CI = [0.2607, 0.5342]) and consequently a lower *P* value by the two-tailed Student's *t*-test (Method 1, *P* = 0.00039; Method 2, *P* = 0.00024).

We also applied the DFT-based temporal frequency filter to a different set of control and PFA-fixed cell image sequences collected at higher temporal resolution to further distinguish actual biological motility from artifact. Filtering cells with a 0.0026 Hz temporal frequency (period < 6.4 min) threshold decreased the already low motility index values of PFA-fixed cells (as measured using Method 2) by 66% on average (Figure 6(e)), confirming the value of the temporal frequency filter for reducing spurious “motility” from measurements. In contrast, the motility index value of the control live cells was only reduced by an average of 23%, consistent with most of the signal being biological in origin. Thus, the temporal frequency filter in conjunction with our improved motility index provides a set of useful and sensitive tools for the detection and quantification of biologically relevant morphological remodeling of cells from 2-photon time-series images. 

## 4. Discussion

We present a novel method for motility analysis of a wide variety of morphologically complex cells. This versatile approach offers increased sensitivity compared to the earlier version due to its superior discrimination of real movement from noise. This is achieved by boxcar averaging of pixel redistribution images, which results in the relatively greater weighting of areas in the images where large groups of adjacent pixels exhibit coordinated changes from one time point to another, while reducing the contribution of spurious pixel fluctuations. When the size of the boxcar window is set to roughly match that of the most relevant motile structure for the phenomenon under study (e.g., dendritic spines, axonal growth cones, and glial filopodia), our algorithm returns a single motility index value that effectively reflects the degree of motility of the specific individual structures of interest without requiring painstaking and time-consuming reconstruction of each of these cellular protuberances. In addition, we demonstrate that further improvement can be obtained by temporal frequency filtering of the time series.

Real biologically relevant movements like filopodial extension typically involve changes of larger groups of neighboring pixels. Changes entailing the redistribution of a smaller number of adjacent pixels from one time point to another often arise from artifacts including photobleaching, detector noise, and imperfect alignment of time series images. This is most evident in the faint outlining of the whole perimeter of the cell in pixel redistribution images (Figure 3(b)). This perimeter outlining is unlikely to be related to cellular metabolism or function, as it is also present in the PFA-fixed cells. The use of boxcar averaging dramatically reduces the contribution of this artifactual component to our motility measurements by weakening the influence of isolated pixel values while enhancing the weighting of clusters of pixels that change together.

The new Method 2 consistently yields lower coefficients of variance than the original Method 1. The principal reason is the above-mentioned reduction of noise at the cell perimeter. In addition, eliminating the need for normalization by cell area removes another source of variability that is not relevant to actual motility. Radial glia cells appear to come in an endless variety of shapes and sizes, and the number of processes does not necessarily linearly scale with the area. Because Method 1 relies on counting the total number of redistributed pixels between time points, it is necessary to normalize these values in order to correct for different cell sizes to prevent larger, less motile cells from being scored higher than highly motile cells that are just very small. However, normalizing to total cell area can result in two cells with identical motility of their processes but different 2D areas being reported as having different motility rates. The use of boxcar averaging in Method 2, in contrast, gives a value that reflects process movement within local domains that is independent of the total size of the cell.

To further eliminate spurious pixels, we have designed a filter based on temporal frequency of the pixel fluctuations. Some of the most rapid and stochastic changes in pixel values occur along the perimeter of imaged cells where out-of-focus light, photon scattering, as well as fine image drift enhance the inherent fluctuations in signal detection at photomultiplier tubes or other detectors. By thresholding out the highest temporal frequency pixels, mainly biologically relevant pixels remain. A surprisingly large proportion (23%) of the prefiltered motility measures of normally active cells appeared to be derived from spurious fluctuations. This also helps explain why the motility index scores of PFA-fixed glial cells were not simply zero, as a considerable fraction of the motility index appears to be derived from nonbiological artifact. Thus, the application of temporal frequency domain filtering has tremendous potential for improving the dynamic range and sensitivity of the motility index. However, temporal frequency filtering can be properly applied only when numerous rapid interval images have been collected. The optimal acquisition rate will depend upon the velocity of the remodeling processes of the cell and on the pixel size of the imaging system. 

The question of what constitutes the remaining “motility” in fixed cells, after the spurious edge pixels have been removed, is intriguing. The images may gradually change due to photobleaching, photochemistry releasing reactive species, and perhaps even thermal effects from the imaging laser. The finding that fixed material exhibits considerable spurious motility raises potential questions about the correct interpretation of the numerous studies reporting continuous subtle movements of processes like dendritic spines or astrocytic filopodia under the microscope. This argues that appropriate fixative-treated controls should be tested as a first step whenever measuring cellular dynamics in order to define the noise floor of the imaging system used.

In certain cases it would be useful to measure motility of only selected parts of a cell. We tested choosing user-defined smaller parts of the images for motility analysis during the course of our algorithm development. However, we ultimately settled on the automatic boxcar-filtered approach for the majority of our analyses because it allows for truly objective quantification of motility without introducing investigator bias, for example, due to selecting specific regions of interest. One interesting future possibility for comparing defined subregions of cells without bias might be to have the software create an internal standard coordinate system for all cells, as was done in a study analyzing bacterial division [20]. Another important future direction would be to implement a similar unbiased motility analysis for the full original three-dimensional data sets, rather than maximum intensity projections. In light of the large amount of noise introduced by image drift and registration over time, this problem will be compounded greatly by adding a three-dimensional registration step, especially complicated given the anisotropy of axial resolution compared with lateral image resolution on most optical microscopes. New high-resolution light-sheet microscopy techniques such as selective plane illumination microscopy (SPIM) and Bessel beam microscopy should facilitate extending these analyses into 3D [21, 22].

## 5. Conclusions

We have created a robust and sensitive method to measure structural motility of any cell type and generate a quantitative motility index. This method is especially useful for cells with complex morphologies that are complicated to analyze by other means. The programs implementing this method with a user-friendly interface are freely available for download at http://ruthazerlab.mcgill.ca/downloads/motility_GUIv2.zip.
